# Relationship between attachment anxiety and attachment avoidance, mentalization ability, and various digital addictions: a cross-sectional study among university students

**DOI:** 10.1186/s12888-026-07776-w

**Published:** 2026-01-23

**Authors:** Lara Antonia Jasper, Henrik Bischoff, Christiane Eichenberg

**Affiliations:** 1 Faculty of Psychology, Sigmund Freud Private University, Vienna, Austria; 2 Faculty of Medicine, Institute of Psychosomatics, Sigmund Freud Private University, Vienna, Austria

**Keywords:** Digital addiction, Attachment, Mentalization ability, Social media addiction, Smartphone addiction

## Abstract

**Background:**

The increasing prevalence of digital media use has been accompanied by a rise in behavioral addictions, such as smartphone and social media addiction. Theoretical models based on attachment theory suggest that insecure attachment styles, particularly attachment anxiety and avoidance, may predispose individuals to maladaptive digital behaviors as a form of affect- and relationship regulation. In addition, mentalization theory posits that the ability to understand one’s own and others’ mental states serves as a protective factor in affect- and relationship regulation. However, little is known about how mentalization ability interacts with attachment insecurities in predicting digital addictions.

**Research question:**

This study examines the relationship between attachment anxiety, attachment avoidance, mentalization ability, and digital addictions among students in Germany and Austria. Specifically, it analyzes whether mentalization ability moderates the relationship between attachment insecurities and digital addictions.

**Methods:**

A cross-sectional study was conducted with *N* = 324 students from Germany and Austria. Participants completed questionnaires assessing their attachment style (ECR-RD), mentalization ability (MZQ-6), and forms of digital addiction (smartphone, social media). Hierarchical multiple regression analyses were conducted to examine main effects and interaction terms, testing moderation models.

**Results:**

Attachment anxiety emerged as a robust predictor of both forms of digital addiction, particularly social media addiction. Attachment avoidance was also significantly associated with digital addiction but to a lesser extent. Crucially, mentalization ability moderated the association between attachment avoidance and social media addiction, such that individuals with high avoidance and low mentalization were most vulnerable.

**Discussion:**

These findings support the integration of attachment theory and mentalization theory in understanding digital addictions. The results highlight that mentalization ability may buffer the adverse effects of attachment avoidance, suggesting that interventions targeting both attachment dynamics and mentalization skills could enhance prevention and treatment efforts. Future studies should further explore this interaction using longitudinal and clinical designs.

## Introduction

### The rise of digital media and its problematic impact on daily life

The pervasive integration of digital media has transformed communication but also raised concerns about mental health. In 2020, the World Health Organization (WHO) acknowledged digital technology addiction as a global health issue [1]. Intensive use can lead to behavioral addictions such as smartphone and social media addiction, characterized by compulsive and dysregulated engagement despite harmful consequences [2–4]. Smartphone addiction typically refers to excessive and uncontrolled use of mobile devices across multiple functions [5], while social media addiction involves compulsive use of networking platforms for interaction, self-presentation, or emotional regulation [6]. These conditions are increasingly recognized as substantial psychological disorders [7, 8], though research is still limited and they are not yet formally classified in DSM-5 or ICD-11 [9].

### Prevalence of digital addictions among young adults

A meta-analysis of 495 articles with over two million participants from 64 countries estimated global prevalence rates of 26.99% for smartphone addiction and 17.42% for social media addiction, making these the most widespread forms of digital addiction. Cybersex (8.23%) and gaming addiction (6.04%) showed lower prevalence rates. Digital addictions have increased steadily over the past two decades, with a marked rise during the COVID-19 pandemic [10]. These findings suggest that smartphone and social media addiction represent widespread and growing public health concerns.

Young adults are particularly vulnerable, as digital media play a central role in both academic and social life during this developmental stage. Identity formation, social comparison, insecure attachment, and emotion regulation challenges contribute to this susceptibility [11–18]. Limited mentalization ability may further amplify risks by impairing reflection on emotional states and social interactions, reinforcing reliance on digital platforms for regulation and connection [19].

Consistent with this, a meta-analysis of 83 samples across 24 countries (*N* = 33,831) documented a steady rise in problematic smartphone use between 2014 and 2020 [20]. Prevalence estimates vary by country and measurement tools, ranging from 36.8% in Nepal [21] to 64.7% in India [22], with lower rates in Serbia (21.7% [23]) and Austria (15.1% [24]) (see also [25–27]). Similarly, a review of 51 studies with 35,520 students reported a pooled prevalence of 18.4% for social media addiction among university students, with the highest rates in Asia (22.8% [28]). These findings illustrate that prevalence is context-dependent and influenced by cultural norms, measurement, and media environments.

Beyond clinically manifest addiction, excessive social media use is linked to anxiety, depression, suicidality, and lower quality of life, with mechanisms such as social comparison, fear of missing out, cyberbullying, and negative feedback playing central roles [29, 30]. Clinically relevant digital addictions are also associated with comorbidities such as mood disturbances, impulsivity, sleep problems, and aggressiveness [31]), as well as reduced physical activity, underscoring their broader public health impact [32]. Despite rising concern, evidence for effective long-term interventions remains limited [33], highlighting the need to examine underlying psychological mechanisms in their development and maintenance.

### Mental health comorbidities linked to digital addictions in young adults

The rise of digital addictions, especially among young adults, highlights the need to understand their associated comorbidities, which are significant in this demographic. These addictions are closely linked to mental health issues like depression, mood disorders, and impulsivity, particularly in young males, and contribute to sleep disturbances and aggressiveness [31] [34, 35]). Smartphone addiction, common among university students, is associated with anxiety, depression, stress, and in severe cases, suicidal thoughts [16, 27, 36, 37]. It also correlates with reduced gray matter volume in the brain, raising concerns about its impact [38]. Social media addiction is prevalent and linked to depression, anxiety, and substance use, with personality traits like loneliness increasing susceptibility [39–41]. These comorbidities underscore the need for targeted research into the correlations and interventions for young adults.

### Attachment theory and its implications for digital media addiction

The smartphone has evolved into a constant companion, providing support and comfort and often functioning as a pseudo-attachment object [42]. Its portability, personal nature, and tactile qualities allow it to serve as an “adult pacifier” [43, 44]. Given these features, attachment theory offers a useful framework for understanding digital media addiction. Prior research shows that insecure attachment styles are associated with behavioral dependencies [45]. A systematic review of 32 studies confirmed that both anxious and avoidant attachment predict social media addiction, as individuals may use online platforms to compensate for unmet needs for affection [46]. Thus, insecure attachment represents a vulnerability factor for digital addictions [9, 24], and therapeutic approaches may benefit from an attachment-focused perspective [47].

Attachment theory, developed by Bowlby [48] and Ainsworth & Bell [49], describes the emotional bonds between children and caregivers, which form the basis for later relationship patterns. These attachment systems shape internal working models that guide adult relationships and tend to remain stable, though they can be modified by later experiences [50, 51].

Different insecure styles have distinct implications for digital behavior. Ambivalently attached individuals may use social media anonymity for self-representation, driven by heightened needs for closeness and social support, which also impairs mentalization [9, 52]. Excessive social media use in this group is linked to suicidality, low self-esteem, and emotion regulation problems [53] and is associated with social anxiety [54].

Problematic smartphone use is likewise related to insecure attachment. Attachment anxiety is associated with smartphone addiction, mediated by loneliness and depression [24, 55]. Avoidant attachment is linked to low self-esteem and anxiety, which also contribute to addictive use [56]. In contrast, strong bonds with parents and peers can reduce problematic use by fostering self-efficacy and prosocial thinking [57]. Young adults with higher attachment anxiety are particularly prone to using smartphones as attachment objects [58].

In social media contexts, attachment anxiety and avoidance are both risk factors for addiction [46, 59, 60]). Anxious attachment increases vulnerability through needs for relatedness and self-presentation, while avoidance reflects motives for autonomy; both are amplified by poor emotion regulation [59]. Anxious attachment, in particular, is strongly linked to excessive social media use, driven by low self-esteem and the pursuit of belonging and relationships [61].

In summary, attachment styles developed in childhood shape digital behavior across the lifespan [46]. Their role in digital addictions underscores the importance of incorporating attachment perspectives into both research and therapeutic interventions.

### Mentalization ability: its potential influence on digital addictions

In relation to attachment style, the psychological concept of mentalization ability is also highly relevant. Mentalization refers to the capacity to recognize and respond to the cognitive and emotional states of oneself and others. Acquired in childhood, it is strongly shaped by early attachment experiences and influences later relationships [62–64]. Secure attachment fosters robust mentalizing, whereas insecure attachment often results in impaired mentalization, which contributes to the development of mental disorders [65, 66]. Intensive media use in childhood can also hinder the development of this ability [67].

Because mentalization is shaped by early attachment, it plays a crucial role in interpersonal functioning and may also be central to digital addictions [67]. Mentalizing processes involve brain regions such as the medial prefrontal cortex, precuneus, and temporoparietal junction, which specialize during childhood and adolescence [68]. These networks, together with the mirror neuron system, support perspective-taking and the representation of self and others [69]. Importantly, the same brain areas are implicated in addictions, including smartphone addiction [70–73].

Thus, impaired mentalization not only increases risk for mental disorders but also for problematic digital behavior. Its relevance is underscored by findings that mentalization deficits are linked to substance-related addictions [74], suggesting a transdiagnostic role across addictive behaviors. Building on prior work connecting insecure attachment to psychological disorders and both substance and behavioral addictions [75–77], this study examines how attachment anxiety, attachment avoidance, and mentalization are related to digital addictions.

While links between insecure attachment and digital addictions have been explored, the role of mentalization remains under-investigated. Individuals with insecure attachment frequently show impaired mentalization, reflecting disrupted early experiences that hinder a stable sense of self and others [62, 63, 65]. Conceptually, mentalizing can be framed as a key psychological factor linking attachment insecurity with problematic digital media use. Impaired mentalizing is associated with difficulties in recognizing one’s own emotions and interpreting social interactions [78], which may increase reliance on digital media for regulation and connection [79]. Digital platforms offer simplified, controllable environments with fewer complex interpersonal cues [80–82], which can be especially attractive for individuals with reduced mentalizing capacity in the absence of satisfying offline relationships [19]. Consequently, excessive digital media use may serve both as compensation for social contact [83, 84] and as a form of emotional escape or avoidance [8]. In this context, we examine whether mentalization ability moderates the relationship between insecure attachment and digital addictions.The following hypotheses and research questions guide this study:

1. Association between insecure attachment and digital addiction
  *Research Question 1 (RQ1):* Are higher levels of attachment anxiety and attachment avoidance associated with increased symptoms of smartphone and social media addiction?
  *Hypothesis 1 (H1):* Individuals reporting higher attachment anxiety and avoidance will show significantly higher levels of smartphone and social media addiction. This expectation is based on previous studies showing robust associations between insecure attachment and problematic digital media use [9, 46, 55, 59]. Smartphone and social media addiction were selected due to their high prevalence, particularly among young adults [10].
  Moreover, prior research suggests that digital addictions, particularly social media and smartphone overuse, may vary by gender [85]. Women tend to report higher levels of problematic social media use and smartphone addiction [86], possibly due to differences in socialization patterns and emotion regulation strategies [87]. In line with these findings, gender differences were also explored in the present study.
2. Moderating role of mentalization ability
  *Research Question 2 (RQ2):* Does mentalization ability moderate the association between insecure attachment and digital addiction?
  *Hypothesis 2 (H2):* Lower mentalization ability will be associated with a stronger relationship between insecure attachment (anxiety/avoidance) and symptoms of digital addiction. This hypothesis draws on research linking impaired mentalizing to attachment insecurity and emotional dysregulation [62, 65, 66], and on findings suggesting a potential role of mentalizing difficulties in maladaptive digital coping strategies [64, 67].
3. Differences between addiction profiles
  *Research Question 3 (RQ3):* Do individuals with multiple digital addictions differ from individuals with only one or no digital addiction in terms of attachment style and mentalization ability?
  *Hypothesis 3 (H3):* Students with both smartphone and social media addiction are expected to exhibit higher levels of attachment anxiety and avoidance, as well as lower mentalization ability, compared to students with only one or no digital addiction. This is based on cumulative risk theories and prior evidence linking multiple behavioral addictions to higher psychosocial vulnerability [41, 61, 77].

## Methodology

### Study design

The present study was designed as a cross-sectional study to analyze the relationship between attachment anxiety and avoidance, mentalization abilities, and digital addictions in students. The cross-sectional design allowed for a comprehensive assessment of the prevalence of digital media addictions and the associated psychological constructs, specifically attachment-related insecurities and mentalization abilities, within the target group at a specific point in time.

### Procedure

Data collection took place between December 2023 and March 2024. Participants were recruited from Sigmund Freud Private University (Vienna, Linz, Berlin) via a multi-channel strategy including university mailing lists, flyers, classroom presentations, and on-site invitations.

The online questionnaire was administered using the SoSci Survey platform (https://www.soscisurvey.de/). All participants received detailed information about the study’s purpose, their rights, and data protection. Participation was voluntary, and informed consent was obtained digitally. Respondents were informed that they could withdraw from the study at any time without consequences.

This study was approved by the Ethics Commission of the Faculty of Psychotherapy Science and the Faculty of Psychology at Sigmund Freud University Vienna (Check digit: YCWYLY3XBVXKX290500).

### Measures

The following survey instruments were used to ensure a comprehensive understanding of the phenomena investigated:

- **Socio-demographic data:** A specially developed sociodemographic query collected basic information such as age, gender, relationship status and stage of study in order to characterize the sample and identify possible demographic influences on the study results.
- **Usage behavior:** Questions on digital media usage behavior were asked in order to obtain a detailed picture of the participants’ digital habits. This included the frequency and intensity of use of various internet platforms as well as the underlying motives for use. Specifically, participants were asked:
  - How much time they spend online per day, both for leisure and for academic/work-related purposes, reported in minutes.
  - How frequently they use specific platforms, including WhatsApp, Instagram, YouTube, TikTok, Snapchat, Pinterest, and X (formerly Twitter), rated on a five-point Likert scale from 1 (“Never”) to 5 (“Very often”).
  - Their primary motives for internet use, such as entertainment and communication, rated on a five-point Likert scale from 1 (“Strongly disagree”) to 5 (“Strongly agree”).
  - Whether they use social media primarily passively or actively, with response options ranging from “mostly passive consumption” to “mostly active participation” (e.g., posting and interacting).
- **Mentalization Questionnaire - Short Scale** (MZQ-6 [88]); was utilized to assess participants’ mentalization ability, which refers to the capacity to understand and interpret one’s own and others’ mental states. The MZQ-6 is a concise, 6-item version of the original 15-item Mentalization Questionnaire [89]. The six items are rated on a five-point Likert scale ranging from 1 (“strongly disagree”) to 5 (“strongly agree”). A higher sum score (ranging from 6 to 30) indicates poorer mentalization ability.
- The MZQ-6 has been validated in a German-speaking cohort and demonstrates good internal consistency (ω = 0.88 [88]). In the present sample, internal consistency was acceptable (Cronbach’s α = 0.76).
- **Experiences in Close Relationships - Revised** (ECR-RD [51]); was used to assess the attachment styles of participants. This 36-item self-report questionnaire, specifically adapted for German contexts, measures the dimensions of “attachment anxiety” and “attachment avoidance” using a 7-point Likert scale. Participants rate each item from 1 (“strongly disagree”) to 7 (“strongly agree”). The ECR-RD advises respondents to reflect on their general relationship experiences rather than focusing on a specific current relationship, making it applicable even for individuals who have not yet had romantic relationship experiences. The psychometric properties of the ECR-RD are strong, with α = 0.91 (attachment anxiety) and α = 0.92 (attachment avoidance [51]). In this study, reliability was also high (α = 0.89 for attachment anxiety, α = 0.90 for attachment avoidance).
- **The Smartphone Addiction Scale** (SPAS [90]) assesses five core symptoms of problematic smartphone use: disregard of harmful consequences, excessive preoccupation with smartphone use, inability to control craving, productivity loss, and anxiety. The original instrument comprises 19 items, each rated on a five-point Likert scale. These items were adapted from existing measures of problematic media use, including the Mobile Phone Problematic Use Scale (MPPUS), the Internet Addiction Test, and the Television Addiction Scale, but together form a single, unified scale rather than separate inventories [90]. In addition to the full symptom scale, Bian and Leung [90] proposed an 8-item screening index based on DSM-IV-equivalent addiction criteria. In the present study, this 8-item index was used to distinguish participants with versus without problematic smartphone use. Items were dichotomized and summed to yield a total score ranging from 0 to 8, with a cut-off score of ≥ 5, following the procedure recommended by the original authors.

Regarding psychometric properties, the authors report an internal consistency of α = 0.70 in their validation study. In the present study, the shortened 8-item version showed good reliability (Cronbach’s α = 0.79).

**Bergen Social Media Addiction Scale** (BSMAS [91]); was used in this study to diagnose social media addiction and helps categorize usage behavior as either dependent or non-dependent. This scale is based on six diagnostic criteria commonly used in measurement instruments [92]: mood modification through social media use, salience, withdrawal symptoms when access to social networks is restricted, relapse after abstinence, tolerance development, as well as interpersonal and intrapsychic conflicts arising from pathological use. All items of the BSMAS refer to individual experiences with social media use within a 12-month period. The BSMAS is a self-report questionnaire consisting of six items, each rated on a five-point Likert scale from 1 (“Never”) to 5 (“Very often”). The total score ranges from 6 to 30, with higher scores indicating a greater degree of social media addiction. A cut-off score of 19 suggests a higher likelihood of addiction. Original studies report α = 0.88 [91]. In our sample, internal consistency was good (α = 0.82).

### Sample characteristics

The study included 324 students from the Sigmund Freud Private University, ranging in age from 18 to 72 years (*M* = 25.27, *SD* = 7.03). Of the participants, 259 identified themselves as female (79.9%), *n* = 56 as male (17.3%), and *n* = 9 as diverse (2.8%). The average time spent online for leisure was 160.61 minutes per day (*SD* = 91.50), while the average time spent online for work or university was 126.75 minutes per day (*SD* = 105.58). Overall, students spent an average of approximately 4.79 hours per day online.

Regarding the stage of study, 59.9% (*n* = 194) of the participants were enrolled in a Bachelor’s program, 38.9% (*n* = 126) were in a Master’s program, and 1.2% (*n* = 4) were pursuing a doctoral degree. In terms of geographic distribution, 11.4% (*n* = 37) of the students were studying in Germany, while the remaining 88.6% (*n* = 287) were studying in Austria.

In terms of relationship status, 33.3% (*n* = 108) of the participants reported being single without a partner, 57.4% (*n* = 186) were in a relationship, 6.2% (*n* = 20) were married, 0.3% (*n* = 1) were widowed, and 1.2% (*n* = 4) chose “Other” as their relationship status Table 1.

**Table 1:** Sample characteristics and descriptive statistics for psychological variables (*N* = 324)

Variable	M	SD	Range
Age (years)	25.27	7.03	18–72
Time Online (Leisure)	160.61	91.5	-
Time Online (Work/Study)	126.75	105.58	-
Attachment Anxiety	2.77	1.21	1.00–6.39
Attachment Avoidance	2.43	1.1	1.00–6.28
Mentalization Ability	15.56	4.88	6.00–29.00
Smartphone Addiction	3.2	1.97	-
Smartphone Addiction (*n* = 44; 13.6%)	-	-	-
Social Media Addiction	13.8	5.02	-
Social Media Addiction (*n* = 57; 17.6%)	-	-	-

Note. Time online is reported in minutes per day. Higher MZQ scores reflect poorer mentalization ability. Smartphone and social media addiction categories reflect the proportion of participants exceeding the respective cutoff. For attachment: ECR-RD = Experiences in Close Relationships – Revised (German Version); MZQ = Mentalization Questionnaire; SPAS = Smartphone Addiction Scale; BSMAS = Bergen Social Media Addiction Scale

### Data analysis

The data analysis proceeded in several steps to examine the relationships between attachment anxiety, attachment avoidance, mentalization ability, and digital addictions (social media, smartphone addiction).

The dataset used for the present analyses included all participants who had completed all relevant measures on attachment, mentalization, and digital addiction (*N* = 324). Prior to analysis, the data were screened for completeness, plausibility, and univariate outliers. Missing data were handled via listwise deletion, and only fully completed cases were retained for the final analysis.

Outliers were defined as standardized values exceeding ±3 standard deviations from the mean. None of the observed values met this criterion or showed undue influence, and therefore no additional cases were excluded.

Normality of continuous variables was assessed using Q-Q plots, histograms, and the Kolmogorov-Smirnov test. Minor deviations from normality were tolerated in view of the large sample size, as parametric methods are robust under such conditions.

### Statistical Evaluation

Following data preparation, a descriptive statistical analysis was conducted to gain a basic understanding of the sample characteristics and distribution of the main variables. This included measures of central tendency (mean) and variability (standard deviation), as well as the creation of frequency distributions for categorical variables. For the inferential statistical analysis, bivariate Pearson correlations were first calculated between the main variables to gain initial insights into possible correlations.

Multiple regression analyses were then conducted to investigate the influence of attachment anxiety, attachment avoidance, and mentalization abilities on the various forms of digital addiction. Potential confounding variables, such as age and gender, were included in the models as control variables. To explore whether mentalization ability acts as a moderator between attachment styles and digital addictions, interaction terms were included in the regression models and analyzed using multiple regression analyses.

Additionally, ANOVAs were performed to examine gender-specific differences in digital addictions, attachment styles, and mentalization abilities. Group comparisons using t-tests for independent samples were conducted to assess differences between various groups, including individuals with single versus multiple digital addictions, as well as those with only one specific addiction (e.g., smartphone addiction) compared to those with additional addictions. Mann-Whitney U-tests were employed when the data did not meet the assumptions of normality.

All statistical analyses were performed using SPSS (IBM, version 29.0.0.0). The selection of specific tests and models was based on the characteristics of the data and the specific research questions.

## Results

### Usage behavior, motives and addiction prevalence

Usage behaviour of digital media in the sample reveals that WhatsApp is the most frequently used application, with 71.6% of participants reporting that they use the platform “very often.” Instagram follows with 44.8% regular usage, while YouTube is “very often” used by 21.9% of respondents. Platforms like TikTok, Snapchat, and Pinterest are used less frequently, with X (formerly Twitter) being the least utilized platform, as 84.0% of respondents indicated they “never” use it.

Regarding daily online time during leisure, 53.4% of respondents reported spending between 120 and 240 minutes online, while 41.7% spend less than 60 minutes online daily for work or study. On average, participants spend a total of about five hours online daily, with approximately 160 minutes dedicated to leisure activities and 125 minutes to work or study.

The main motives for internet use include entertainment (59.3% “strongly agree”) and interaction and communication (64.5%). A passive use of social media platforms is preferred by 44.8% of respondents, while only 4.6% actively engage. This suggests a predominantly consumptive behavior, with relatively low active participation in social networks. These findings provide important insights into the preferences and habits of internet usage within the sample.

### Attachment anxiety and attachment avoidance

For attachment avoidance, the mean score was 2.43 (*SD* = 0.10), with a range from 1.00 to 6.28. For attachment anxiety, the mean score was 2.77 (*SD* = 1.21), with scores ranging from 1.00 to 6.39. These results place the sample within the normative range, as the normative sample had mean scores of 2.92 (*SD* = 1.19) for avoidance and 3.56 (*SD* = 1.12) for anxiety [51].

### Mentalization ability

The sample had a mean score of 15.56 (*SD* = 4.88), with scores ranging from 6.00 to 29.00. Compared to a normative sample with a mean score of 13.2 (*SD* = 4.66), the present sample scored slightly higher, indicating poorer mentalization abilities, as higher scores reflect worse performance in this area [88].

### Smartphone addiction

The mean score for smartphone addiction was 3.20 (*SD* = 1.97). Within the sample, 13.6% (*n* = 44) were identified as dependent smartphone users, while 86.1% (*n* = 279) were categorized as non-dependent.

### Social media addiction

The mean score for social media addiction was 13.80 (*SD* = 5.02). The analysis identified 17.6% (*n* = 57) of participants as having an addictive use of social media, with the remaining 82.1% (*n* = 266) classified as non-addictive users.

### Attachment style and digital addictions

The results showed that higher scores in attachment anxiety and attachment avoidance were associated with an increased risk of digital addiction. In particular, attachment anxiety showed a strong positive correlation with social media addiction (*r* = 0.46, 95% CI [0.37, 0.54], *p* < 0.001) and smartphone addiction (*r* = 0.34, 95% CI [0.24, 0.43], *p* < 0.001). Avoidance of attachment also correlated positively with digital addictions, albeit to a lesser extent (smartphone addiction: *r* = 0.14, 95% CI [0.03, 0.24], *p* < 0.05 and social media addiction: *r* = 0.24, 95% CI [0.13, 0.34], *p* < 0.001).

### Mentalization ability and digital addictions

A lower ability to mentalize correlates positively with the digital addictions investigated, meaning that as mentalization ability decreases, the severity of digital addictions increases. Significant negative correlations were found with smartphone addiction (*r* = 0.29, 95% CI [0.38, 0.19], *p* < 0.001) and social media addiction (*r* = 0.39, 95% CI [0.47, 0.29], *p* < 0.001).

### Group differences: single vs. Multiple addictions

To further investigate whether individuals with multiple digital addictions differ in relevant psychological characteristics from those with only one form of addiction, independent samples t-tests were conducted.

Individuals with multiple addictions reported significantly higher attachment anxiety (*M* = 0.05, *SD* = 1.21) than individuals with a single addiction (*M* = −0.60, *SD* = 0.97), *t*(30.72) = 3.16, *p* **=** 0.004, *d* = 0.55. No significant differences were observed for attachment avoidance (*M_multiple* = 0.02, *SD* = 1.00; *M_single* = −0.16, *SD* = 0.96), *t*(28.57) = 0.89, *p* = 0.382, *d* = 0.18.

In contrast, participants with multiple addictions showed significantly lower mentalization ability (*M* = −0.20, *SD* = 4.81) compared to those with a single addiction (*M* = 2.60, *SD* = 5.00), *t*(27.86) = −2.70, *p* = 0.012, *d* = 0.58. These findings support the assumption that psychological vulnerability is increased in individuals affected by more than one form of digital addiction.

### Gender-specific differences

There were significant gender differences in both smartphone addiction, *F*(2, 320) = 5.10, *p* = 0.007, η^2^ = 0.031, and social media addiction, *F*(2, 320) = 13.41, *p* < 0.001, η^2^ = 0.077. Post hoc tests revealed that female participants reported significantly higher levels of smartphone addiction (*M* = 3.36, *SD* = 1.96) than male participants (*M* = 2.44, *SD* = 1.84), *p* = 0.007. For social media addiction, women also scored significantly higher (*M* = 14.32, *SD* = 5.07) compared to men (*M* = 11.40, *SD* = 4.39), *p* < 0.001. Participants identifying as diverse did not significantly differ from male or female participants in either addiction category (all *p* > 0.80).

No significant gender differences were observed for attachment anxiety, *F*(2, 321) = 2.33, *p* = 0.114, η^2^ = 0.014, attachment avoidance, *F*(2, 321) = 0.21, *p* = 0.811, η^2^ = 0.001, or mentalization ability, *F*(2, 321) = 2.02, *p* = 0.151, η^2^ = 0.012 Table 2.

**Table 2:** Correlation matrix of key study variables

Variable	1. BANG	2. BVER	3. MZQ	4. SPAS	5. BSMAS	M (SD)
1. Attachment Anxiety (BANG)						2.77 (1.21)
2. Attachment Avoidance (BVER)	0.36***					2.43 (0.10)
3. Mentalization (MZQ)	−0.28***	−0.22***				15.56 (4.88)
4. Smartphone Addiction (SPAS)	0.34***	0.14*	0.29***			3.20 (1.97)
5. Social Media Addiction (BSMAS)	0.46***	0.24***	0.39***	0.65***		13.80 (5.02)

*Note.* BANG = Attachment Anxiety, BVER = Attachment Avoidance, MZQ = Mentalization (higher = worse), SPAS = Smartphone Addiction, BSMAS = Social Media Addiction

*p < 0.05, **p < 0.01, ***p < 0.001

### Regression analyses

In the study presented here, two separate regression analyses were conducted to examine the relationship between attachment anxiety, attachment avoidance, mentalization ability, and various forms of digital addiction. The results are presented in detail below.

**Fig. 1:**
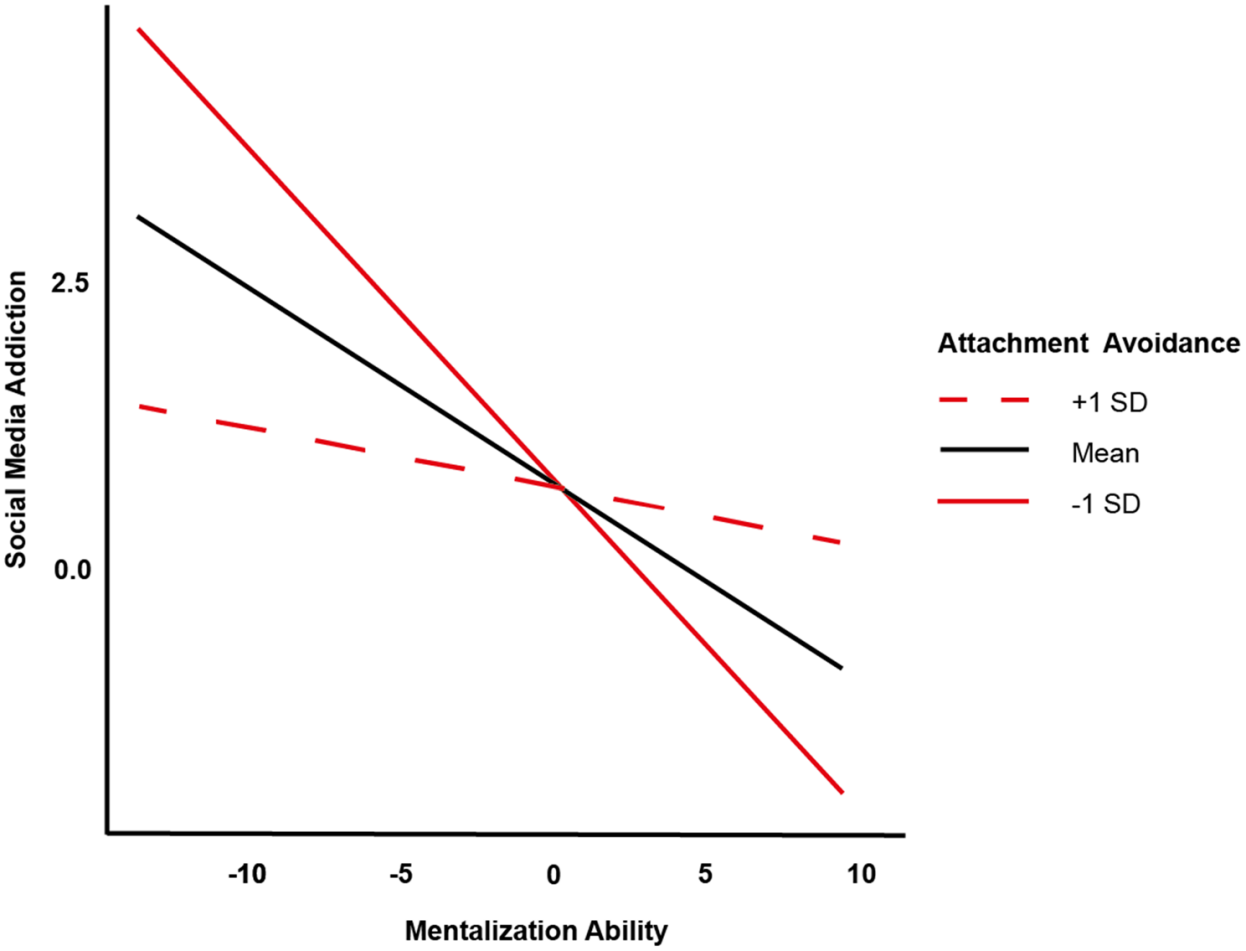
Interaction between mentalization ability and attachment avoidance in predicting social media addiction *note.* the solid black line represents the predicted social media addiction scores at the mean level of attachment avoidance. The solid red line represents predicted social media addiction scores at one standard deviation below the mean of attachment avoidance (low attachment avoidance), while the dashed red line represents predicted social media addiction scores at one standard deviation above the mean of attachment avoidance (high attachment avoidance). The x-axis represents mentalization ability, with higher scores indicating greater mentalization ability. The y-axis represents the predicted social media addiction scores. The interaction suggests that for individuals with low attachment avoidance, an increase in mentalization ability is associated with a sharper decrease in social media addiction. In contrast, for individuals with high attachment avoidance, mentalization ability has a less pronounced effect on reducing social media addiction

With regard to **social media addiction**, the regression analysis revealed significant main effects for gender (*β* = −1.74, 95%-CI [−2.69, −0.79], *p* < 0.001), age (*β* = −0.12, 95%-CI [−0.20, −0.04], *p* = 0.002), attachment anxiety (*β* = 1.50, 95%-CI [0.97, 2.03], *p* < 0.001), and mentalization ability (*β* = −0.18, 95%-CI [−0.30, −0.07], *p* = 0.002). The significant interaction effect between mentalization ability and attachment avoidance (*β* = 0.12, 95%-CI [0.01, 0.23], *p* = 0.031) shows that although social media addiction generally decreases with increasing mentalization ability, this decrease is flatter in people with high attachment avoidance compared to people with lower attachment avoidance (see Fig. 1). This indicates a differentiated relationship between these variables, with mentalization ability having a less pronounced mitigating effect on social media addiction with greater attachment avoidance. The interaction between mentalization ability and attachment anxiety (*β* = 0.01, 95%-CI [−0.09, 0.12], *p* = 0.773) was not significant. The regression model was statistically significant overall (*F*(7, 315) = 20.343, *p* < 0.001, *R*^*2*^* adjusted* = 0.297).

In the regression analysis for **smartphone addiction**, gender (*β* = −0.53, 95%-CI [−0.95, −0.12], *p* = 0.012), age (*β* = −0.06, 95%-CI [−0.09, −0.03], *p* < 0.001), and attachment anxiety (*β* = 0.43, 95%-CI [0.21, 0.65], *p* < 0.001) proved to be significant predictors. Mentalization ability showed no significant influence (*β* = −0.04, *p* = 0.076). A trend emerged in the interaction between mentalization ability and attachment avoidance (*β* = 0.04, 95%-CI [−0.09, 0.00], *p* = 0.066), suggesting that higher mentalization ability in combination with attachment avoidance could potentially influence the risk of smartphone addiction, although this effect was not statistically significant. The interaction between mentalization ability and attachment anxiety (*β* = −0.02, 95%-CI [−0.06, 0.03], *p* = 0.436) was also not significant. The overall model was statistically significant (*F*(7, 315) = 11.504, *p* < 0.001, *R*^*2*^* adjusted* = 0.188) Table 3.

**Table 3:** Hierarchical regression analyses predicting social media and smartphone addiction

	Social Media Addiction			Smartphone Addiction		
Predictor	β (SMA)	95% CI (SMA)	*p* (SMA)	β (SPA)	95% CI (SPA)	*p* (SPA)
Gender (0 = female, 1 = male)	−1.74***	[−2.69, −0.79]	< 0.001	−0.53*	[−0.95, −0.12]	0.012
Age	−0.12**	[−0.20, −0.04]	0.002	−0.06***	[−0.09, −0.03]	< 0.001
Attachment Anxiety	1.50***	[0.97, 2.03]	< 0.001	0.43***	[0.21, 0.65]	< 0.001
Attachment Avoidance	0.24*	[0.13, 0.34]	< 0.001	0.14*	[0.03, 0.24]	0.012
Mentalization Ability	−0.18**	[−0.30, −0.07]	0.002	−0.04		0.076
Mentalization × Avoidance	0.12*	[0.01, 0.23]	0.031	0.04†	[−0.09, 0.00]	0.066
Mentalization × Anxiety	0.01	[−0.09, 0.12]	0.773	−0.02	[−0.06, 0.03]	0.436
Model Summary	F(7, 315) = 20.343***, Adj. R^2^ = 0.297			F(7, 315) = 11.504***, Adj. R^2^ = 0.188		

Note. SMA = Social Media Addiction; SPA = Smartphone Addiction. †*p* < 0.10, *p < 0.05, **p < 0.01, ***p < 0.001. Higher mentalization scores reflect poorer ability

## Discussion

Summary. This study investigated the relationship between attachment anxiety, attachment avoidance, mentalization ability, and digital addictions (smartphone and social media addiction). The prevalence of addiction in the present sample was 13.6% for smartphone addiction and 17.6% for social media addiction. Compared to global prevalence estimates reported by Meng et al. [10], which found 26.99% for smartphone addiction and 17.42% for social media addiction, the prevalence of smartphone addiction in this sample was lower. However, the prevalence of social media addiction was within the range of global estimates and largely consistent with findings from other studies. Notably, the observed prevalence also aligns with the expected range for the Bergen Social Media Addiction Scale (BSMAS), for which Chen et al. [93] report normative prevalence rates between < 10 and 40%, depending on population and cut-off criteria. The observed usage behaviors, such as the high frequency of WhatsApp and Instagram use and the significant daily online time [13], highlight the pervasive role of digital media in the participants’ lives.

Consistent patterns emerged in the regression analyses conducted to investigate the relationship between attachment characteristics, mentalization skills, and digital addictions. Significant predictors for both forms of digital addictions, smartphone addiction and social media addiction, included gender, age, and attachment anxiety, with mentalization ability being particularly significant for social media addiction. An interesting finding was the significant interaction effect between mentalization ability and attachment avoidance in social media addiction, indicating a complex dynamic between these variables. A similar trend in the interaction between mentalization ability and attachment avoidance was observed for smartphone addiction, although this was not statistically significant.

In addition to correlational and regression-based findings, group comparisons between individuals with single versus multiple digital addictions further substantiated the psychological differentiation between risk profiles. Participants with multiple addictions exhibited significantly higher levels of attachment anxiety and significantly lower mentalization ability compared to those with only one form of addiction. These group-level differences suggest that cumulative addictive behaviors may be associated with heightened psychological vulnerability, particularly in terms of emotion regulation and relational insecurity. No significant difference emerged for attachment avoidance, indicating that this dimension may be less sensitive to the additive effect of multiple digital dependencies. Together, these findings reinforce the importance of considering comorbidity within digital addictions when assessing psychological risk factors.

Comparison with previous research. These findings align with previous research showing that attachment anxiety is a robust predictor of digital addictions, particularly in relation to social media use [60, 94]. Individuals with high attachment anxiety may increasingly seek affection and closeness in online interactions, thereby increasing their risk of developing digital addictions [56]. Attachment avoidance was also associated with digital addictions, although the observed effect sizes were smaller. This is consistent with studies suggesting that avoidantly attached individuals might use digital media to maintain a sense of connectedness while minimizing direct social engagement [95]. However, previous findings on the role of attachment avoidance have been inconsistent [96, 97], and the current findings suggest that this dimension may play a role in a more complex interplay with other factors.

A significant relationship between lower mentalization ability and social media addiction supports existing literature suggesting that reduced mentalizing may lead individuals to rely more heavily on digital media for emotion regulation [94]. Moreover, the observed moderation effect indicates that high levels of mentalization ability can buffer against the impact of attachment avoidance on social media addiction, whereas individuals with lower mentalizing capacity may be more vulnerable. This pattern was echoed by a similar, though nonsignificant, interaction in smartphone addiction.

This moderating role of mentalization in the context of avoidant attachment is consistent with theoretical models that suggest individuals with high avoidance may engage in digital behaviors to maintain emotional distance while satisfying a need for indirect social contact [66]. Recent studies have proposed that high mentalization capacity may buffer maladaptive coping strategies in avoidantly attached individuals [94, 98]. While our finding for smartphone addiction only showed a statistical trend, the directionality mirrors that observed for social media addiction and may reflect a broader regulatory mechanism in the digital domain.

Gender differences were also observed: female participants reported significantly higher levels of both smartphone and social media addiction. This replicates existing findings suggesting greater vulnerability to problematic digital media use among women [94]. However, no significant gender differences were found for attachment dimensions or mentalization ability, which diverges from prior studies reporting higher attachment anxiety in women and greater attachment security in men.

These discrepancies might partly reflect cultural influences. Our sample consisted mainly of Austrian and German students, where social media use is embedded in an individualistic cultural context that emphasizes self-presentation and peer comparison. In contrast, studies conducted in collectivistic contexts (e.g., Asia) often report different patterns of gender and attachment effects, likely reflecting distinct social norms and expectations about online interaction.

Furthermore, our findings revealed that mentalization ability was a significant moderator in the case of social media addiction, but not for smartphone addiction. This discrepancy may be explained by the inherently social nature of social media use, which requires individuals to interpret others’ mental states, manage feedback, and navigate online social relationships. In contrast, smartphone use is more heterogeneous and often involves non-social activities (e.g., gaming, browsing, functional use), where mentalizing processes play a smaller role. Thus, the moderating role of mentalization may be specific to contexts in which digital behavior is strongly tied to interpersonal interaction.

Taken together, the results underscore the relevance of attachment anxiety, attachment avoidance, and mentalization ability in understanding the development of digital addictions. The role of mentalization as both a direct predictor and a moderator highlights its potential as a protective factor and suggests that interventions targeting mentalizing skills may be beneficial. Future studies should further investigate the interactive mechanisms between attachment behavior, emotion regulation, and digital media use in longitudinal or experimental designs to clarify causal directions.

## Limitations

While this study provides valuable insights into the relationship between attachment anxiety, attachment avoidance, mentalization ability, and digital addictions among university students, several limitations must be acknowledged. First, the cross-sectional design of the study limits the ability to infer causality between the variables. Although significant associations were found, the directionality of these relationships cannot be conclusively determined, and it remains unclear whether attachment and mentalization issues lead to digital addictions or if these addictions exacerbate attachment and mentalization difficulties.

Second, the study relies on self-reported data, which is susceptible to biases such as social desirability and recall bias. Participants may have underreported or overreported their digital addiction levels or attachment-related behaviors, potentially affecting the accuracy of the findings.

Third, the sample is relatively homogenous, consisting predominantly of psychology students from a specific university in Austria and Germany. This limits the generalizability of the findings to other populations, particularly those from different cultural backgrounds or academic disciplines. The overrepresentation of female participants further constrains the applicability of the results across genders.

Fourth, the study does not account for other potential confounding variables, such as personality traits or existing mental health conditions, which could influence the relationships examined.

Fifth, the study’s focus on only two forms of digital addiction (smartphone and social media addiction) may overlook other relevant digital behaviors or addictions, such as gaming or online gambling, which could also be linked to attachment and mentalization issues. Future research should aim to address these limitations by employing longitudinal designs, diversifying sample populations, and exploring additional digital behaviors and psychological factors.

Finally, although the current study followed a theory-driven moderation approach to test specific interaction effects (mentalization × attachment), alternative multivariate methods such as partial correlation analysis or network modeling may offer additional insights into the complex interrelations among psychological variables. These methods are particularly suited for exploratory, data-driven investigations and may complement hypothesis-driven approaches in future research.

## Outlook and implications

The findings of this study highlight the intricate relationships between attachment styles, mentalization ability, and digital addictions, underscoring the importance of these psychological constructs in understanding and addressing problematic digital behaviors. As digital media continues to permeate daily life, especially among younger populations, it is crucial to consider these factors in both preventative measures and therapeutic interventions. Future research should focus on longitudinal studies to explore the causal pathways between attachment insecurity, mentalization deficits, and digital addiction. Such studies could provide more definitive insights into how these relationships develop over time and inform the creation of targeted interventions.

The implications of this research extend beyond academia, offering practical guidance for mental health professionals, educators, and policymakers. Integrating attachment-focused therapy and mentalization-based interventions could enhance treatment outcomes for individuals struggling with digital addictions. Moreover, educational programs aimed at improving mentalization abilities and fostering secure attachment relationships from an early age could serve as preventative strategies, potentially mitigating the risk of developing digital addictions later in life.

## Conclusion

This study has provided important insights into the complex interplay between attachment anxiety, attachment avoidance, mentalization ability, and forms of digital addictions among university students. The findings suggest that attachment anxiety is a strong predictor of digital addictions, particularly social media addiction, while attachment avoidance shows a more nuanced relationship with these behaviors. Additionally, the study highlights the significant role of mentalization ability, particularly in moderating the relationship between attachment avoidance and social media addiction, suggesting that those with better mentalization abilities may be better equipped to manage their digital media use.

Furthermore, the distinction between single and multiple addictions revealed that individuals with multiple digital addictions exhibit significantly higher levels of attachment anxiety, emphasizing the need for more targeted interventions for this group. The absence of differences in mentalization ability and attachment avoidance between single and multiple addictions suggests that these factors may influence digital addictions in more context-dependent ways, further underscoring the complexity of these relationships.

These results underscore the necessity of considering psychological factors such as attachment styles and mentalization abilities in understanding the development and persistence of digital addictions. They also point to the potential benefits of incorporating these factors into therapeutic interventions aimed at addressing problematic digital behaviors. However, the study’s limitations, including its cross-sectional design, reliance on self-reported data, and the homogeneity of the sample, indicate the need for further research to confirm and extend these findings.

## Data Availability

The datasets generated during and/or analyzed during the current study are available from the corresponding author on reasonable request. All data provided will be anonymized to maintain participant confidentiality in compliance with the ethics approval guidelines.

## Abbreviations

ECR-RD: Experiences in Close Relationships—Revised (Attachment Style Assessment)
MZQ-6: Mentalization Questionnaire Short Scale
SPAS: Smartphone Addiction Scale
BSMAS: Bergen Social Media Addiction Scale

## References

[1] Dresp-Langley B, Hutt A. Digital addiction and sleep. Int J Environ Res Public Health. 2022;19(11):6910. 10.3390/ijerph19116910. 35682491 10.3390/ijerph19116910 PMC9179985

[2] Gonçalves LL, Nardi AE, King ALS. Digital dependence in the past decade: a systematic review. J Addict Res Adolesc Behav. 2023;6(1). 10.31579/2688-7517/059.

[3] Grant JE, Potenza MN, Weinstein A, Gorelick DA. Introduction to behavioral addictions. Am J Drug Alcohol Abuse. 2010;36(5):233–41. 10.3109/00952990.2010.491884. 20560821 10.3109/00952990.2010.491884 PMC3164585

[4] Small GW, Lee J, Kaufman A, Jalil J, Siddarth P, Gaddipati H, et al. Brain health consequences of digital technology use. Dialogues In Clin Neurosci. 2020;22(2):179–87. 10.31887/DCNS.2020.22.2/gsmall. 10.31887/DCNS.2020.22.2/gsmall PMC7366948 32699518

[5] Liu X, Tian R, Liu H, Bai X, Lei Y. Exploring the impact of smartphone addiction on risk decision-making behavior among college students based on fNIRS technology. Brain Sci. 2023;13(9):1330. 10.3390/brainsci13091330. 37759931 10.3390/brainsci13091330 PMC10526789

[6] Cheng C, Ebrahimi OV, Luk JW. Heterogeneity of prevalence of social media addiction across multiple classification schemes: latent profile analysis. J Med Internet Res. 2022;24(1):e27000. 10.2196/27000. 35006084 10.2196/27000 PMC8787656

[7] Bottel L, Brand M, Dieris-Hirche J, Pape M, Herpertz S, Wildt BT. Predictive power of the DSM-5 criteria for internet use disorder: a CHAID decision-tree analysis. Front Phychol. 2023;14, Article 1129769. 10.3389/fpsyg.2023.1129769. 10.3389/fpsyg.2023.1129769 PMC9994355 36910812

[8] Zubair U, Khan MK, Albashari M. Link between excessive social media use and psychiatric disorders. Ann Med Surg. 2023;85(4):875–78. 10.1097/MS9.0000000000000112. 10.1097/MS9.0000000000000112 PMC10129173 37113864

[9] Eichenberg C, Schneider R. Digitale Mediensüchte: Zusammenhang zwischen Bindungsstil und Internet-, Social-Media- und Smartphonesucht. In: Brisch KH, editor. Gestörte Bindungen in digitalen Zeiten: Ursachen, Prävention, Beratung und Therapie. Klett-Cotta; 2023. p. 13–30.

[10] Meng SQ, Cheng JL, Li YY, Yang XQ, Zheng JW, Chang XW, et al. Global prevalence of digital addiction in the general population: a systematic review and meta-analysis. Clin Phychol Rev. 2022;92, Article 102128. 10.1016/j.cpr.2022.102128. 10.1016/j.cpr.2022.102128 35150965

[11] Carcelén-García S, Narros-González MJ, Galmes-Cerezo M. Digital vulnerability in young people: gender, age and online participation patterns. Int J Adolesc Youth. 2023;28(1). 10.1080/02673843.2023.2287115.

[12] Dawson AE, Allen JP, Marston EG, Hafen CA, Schad MM. Adolescent insecure attachment as a predictor of maladaptive coping and externalizing behaviors in emerging adulthood. Attach Hum Devel. 2014;16(5):462–78. 10.1080/14616734.2014.934848. 10.1080/14616734.2014.934848 PMC4146682 24995478

[13] Eichenberg C, Schneider R, Rumpl H. Social media addiction: associations with attachment style, mental distress, and personality. BMC Psychiatry. 2024;24(1):278. 10.1186/s12888-024-05709-z. 38622677 10.1186/s12888-024-05709-z PMC11017614

[14] Kuss D, Kristensen A, Lopez-Fernandez O. Internet addictions outside of Europe: a systematic literature review. Comput In Hum Behav. 2021;115, Article 106621. 10.1016/j.chb.2020.106621.

[15] Nazari A, Hosseinnia M, Torkian S, et al. Social media and mental health in students: a cross-sectional study during the COVID-19 pandemic. BMC Psychiatry. 2023;23, Article 458. 10.1186/s12888-023-04859-w. 10.1186/s12888-023-04859-w PMC10286331 37349682

[16] Okasha T, Saad A, Ibrahim I, Elhabiby M, Khalil S, Morsy M. Prevalence of smartphone addiction and its correlates in a sample of Egyptian university students. Int J Soc Psychiatry. 2021;68:1580–88. 10.1177/00207640211042917. 34479450 10.1177/00207640211042917

[17] Sala A, Porcaro L, Gómez E. Social media use and adolescents’ mental health and well-being: an umbrella review. Comput In Hum Behav Rep. 2024;14:100404. 10.1016/j.chbr.2024.100404.

[18] Wacks Y, Lin Et AM. Excessive smartphone use is associated with health problems in adolescents and young adults. Front Psychiatry. 2021;12, Article 669042. 10.3389/fpsyt.2021.669042. 10.3389/fpsyt.2021.669042 PMC8204720 34140904

[19] Chatterjee D, Rai R. Behind the screens: proposing a mentalization-based theoretical model of problematic Internet use. Cyberpsychol: J Psychosocial Res On Cyberspace. 2023;17(5), Article 6. 10.5817/CP2023-5-6.

[20] Olson J, Sandra D, Colucci É, Bikaii A, Chmoulevitch D, Nahas J, et al. Smartphone addiction is increasing across the world: a meta-analysis of 24 countries. Comput In Hum Behav. 2022;129, Article 107138. 10.31234/osf.io/fsn6v.

[21] Karki S, Singh J, Paudel G, Khatiwada S, Timilsina S. How addicted are newly admitted undergraduate medical students to smartphones?: a cross-sectional study from Chitwan medical college, Nepal. BMC Psychiatry. 2020. 10.1186/s12888-020-02507-1. 32122328 10.1186/s12888-020-02507-1 PMC7052978

[22] Shakya M, Chauhan A, Sharma S, Saraswat A, Rure D, Singh R, et al. Prevalence of smartphone addiction and its association with impulsivity among undergraduate medical students. J Popul Ther Clin Pharmacol. 2023;30(17):1190–98. 10.53555/jptcp.v30i17.2726.

[23] Nikolic A, Bukurov B, Kocic I, Vukovic M, Ladjevic N, Vrhovac M, et al. Smartphone addiction, sleep quality, depression, anxiety, and stress among medical students. Front Public Health. 2023;11, Article 1252371. 10.3389/fpubh.2023.1252371. 10.3389/fpubh.2023.1252371 PMC10512032 37744504

[24] Eichenberg C, Schott M, Schroiff A. Problematic smartphone use-comparison of students with and without problematic smartphone use in light of personality. Front Psychiatry. 2021;11:599241. 10.3389/fpsyt.2020.599241. 33584367 10.3389/fpsyt.2020.599241 PMC7876085

[25] Alotaibi M, Fox M, Coman R, Ratan Z, Hosseinzadeh H. Smartphone addiction prevalence and its association on academic performance, physical health, and mental well-being among university students in umm Al-qura university (UQU), Saudi Arabia. Int J Environ Res Public Health. 2022;19(6), Article 3710. 10.3390/ijerph19063710. 10.3390/ijerph19063710 PMC8954621 35329397

[26] Tangmunkongvorakul A, Musumari P, Tsubohara Y, Ayood P, Srithanaviboonchai K, Techasrivichien T, et al. Factors associated with smartphone addiction: a comparative study between Japanese and Thai high school students. PLoS One. 2020;15, Article e0238459. 10.1371/journal.pone.0238459. 10.1371/journal.pone.0238459 PMC7478618 32898191

[27] Zhong Y, Ma H, Liang Y, Liao C, Zhang C, Jiang W. Prevalence of smartphone addiction among Asian medical students: a meta-analysis of multinational observational studies. Int J Soc Psychiatry. 2022;68:1171–83. 10.1177/00207640221089535. 35422151 10.1177/00207640221089535

[28] Salari N, Zarei H, Hosseinian-Far A, Rasoulpoor S, Shohaimi S, Mohammadi M. The global prevalence of social media addiction among university students: a systematic review and meta-analysis. J Public Health (Berl.). Advance online publication. 2023. 10.1007/s10389-023-02012-1.

[29] Dam VAT, Dao NG, Nguyen DC, Vu TMT, Boyer L, Auquier P, et al. Quality of life and mental health of adolescents: relationships with social media addiction, fear of missing out, and stress associated with neglect and negative reactions by online peers. PLoS One. 2023;18(6):e0286766. 10.1371/journal.pone.0286766. 37285351 10.1371/journal.pone.0286766 PMC10246797

[30] Weigle PE, Shafi RMA. Social media and Youth mental health. Curr Psychiatry Rep. 2024;26(1):1–8. 10.1007/s11920-023-01478-w. 38103128 10.1007/s11920-023-01478-w

[31] Diotaiuti P, Mancone S, Corrado S, Risio A, Cavicchiolo E, Girelli L, et al. Internet addiction in young adults: the role of impulsivity and codependency. Front Psychiatry. 2022;13, Article 893861. 10.3389/fpsyt.2022.893861. 10.3389/fpsyt.2022.893861 PMC9485605 36147985

[32] Nambirajan MK, Vidusha K, Kailasam JG, Kannan S, Govindan D, Ganesh K, et al. Association between smartphone addiction and sedentary behaviour amongst children, adolescents and young adults: a systematic review and meta-analysis. J Psychiatric Res. 2025;184:128–39. 10.1016/j.jpsychires.2024.12.018. 10.1016/j.jpsychires.2025.02.049 40049119

[33] Lu P, Qiu J, Huang S, Wang X, Han S, Zhu S, et al. Interventions for digital addiction: umbrella review of meta-analyses. J Med Internet Res. 2025;27(1):e59656. 10.2196/59656. 39933164 10.2196/59656 PMC11862776

[34] Gao T, Li M, Hu Y, Qin Z, Cao R, Mei S, et al. When adolescents face both internet addiction and mood symptoms: a cross-sectional study of comorbidity and its predictors. Psychiatry Res. 2020;284, Article 112795. 10.1016/j.psychres.2020.112795. 10.1016/j.psychres.2020.112795 31986358

[35] Marin M, Núñez X, Almeida R. Internet addiction and attention in adolescents: a systematic review. Cyberpsychol Behav Soc Netw. 2020. 10.1089/cyber.2019.0698. 10.1089/cyber.2019.0698 33121255

[36] Boumosleh J, Jaalouk D. Depression, anxiety, and smartphone addiction in university students: a cross-sectional study. PLoS One. 2017;12, Article e0182239. 10.1371/journal.pone.0182239. 10.1371/journal.pone.0182239 PMC5544206 28777828

[37] Sohn S, Rees P, Wildridge B, Kalk N, Carter B. Prevalence of problematic smartphone usage and associated mental health outcomes amongst children and young people: a systematic review, meta-analysis and GRADE of the evidence. BMC Psychiatry. 2019;19, Article 356. 10.1186/s12888-019-2350-x. 10.1186/s12888-019-2350-x PMC6883663 31779637

[38] Horvath J, Mundinger C, Schmitgen M, Wolf N, Sambataro F, Hirjak D, et al. Structural and functional correlates of smartphone addiction. Addict Behav. 2020;105, Article 106334. 10.1016/j.addbeh.2020.106334. 10.1016/j.addbeh.2020.106334 32062336

[39] Alfaya M, Abdullah N, Alshahrani N, Alqahtani A, Algethami M, Qahtani A, et al. Prevalence and determinants of social media addiction among medical students in a selected university in Saudi Arabia: a cross-sectional study. Healthcare. 2023;11(10), Article 1370. 10.3390/healthcare11101370. 10.3390/healthcare11101370 PMC10217812 37239655

[40] Esfahani M, Niknafs A, Kuss D, Nilashi M, Afrough S. Social media addiction: applying the DEMATEL approach. Telematics Inf. 2019;43, Article 101250. 10.1016/J.TELE.2019.101250.

[41] Szczygieł K, Podwalski P. Comorbidity of social media addiction and other mental disorders - an overview. Archiv Psychiatry Psychotherapy. 2020. 10.12740/app/122487.

[42] Strittmatter E, Greschner M, Müller J, Kaess M, Romer G. Bindung und pathologischer Internetgebrauch [Abstract]. Suchttherapie. 2015;16(1):29. 10.1055/s-0035-1557608.

[43] Carolus A, Binder J, Muench R, Schmidt C, Schneider F, Buglass S. Smartphones as digital companions: characterizing the relationship between users and their phones. New Media Soc. 2018;21:914–38. 10.1177/1461444818817074.

[44] Melumad S, Pham M. The smartphone as a pacifying technology. J Consum Res. 2020;47:237–55. 10.1093/jcr/ucaa005.

[45] Estévez A, Jauregui P, Sánchez-Marcos I, López-González H, Griffiths M. Attachment and emotion regulation in substance addictions and behavioral addictions. J Behav Addict. 2017;6:534–44. 10.1556/2006.6.2017.086. 10.1556/2006.6.2017.086 PMC6034944 29280395

[46] D’Arienzo M, Boursier V, Griffiths MD. Addiction to social media and attachment styles: a systematic literature review. Int J Ment Health Addict. 2019;17:1094–118. 10.1007/s11469-019-00082-5.

[47] Coffey K. The relationship between attachment and addiction. In: Coffey K, editor. The psychology of addiction: an introduction. Springer; 2018. p. 73–79. 10.1007/978-3-319-72778-3_5.

[48] Bowlby J. Attachment and loss: vol. 1 attachment. Basic Books; 1969.

[49] Ainsworth MD, Bell SM. Attachment, exploration, and separation: illustrated by the behavior of one-year-olds in a strange situation. Child Devel. 1970;41(1):49–67. 5490680

[50] De Sanctis F, Mesurado B. Attachment style and empathy in late children, adolescents, and adults: meta-analytic review. Int J Psychological Res. 2022;15(2):114–29. 10.21500/20112084.5409. 10.21500/20112084.5409 PMC10233954 37274515

[51] Ehrenthal JC, Dinger U, Lamla A, Funken B, Schauenburg H. Evaluation der deutschsprachigen Version des Bindungsfragebogens “Experiences in Close Relationships-Revised” (ECR−RD) [Evaluation of the German version of the attachment questionnaire “experiences in close relationships-Revised” (ECR−RD)]. PPmP: Psychotherapie Psychosomatik Medizinische Psychologie. 2009;59(6):215–23. 10.1055/s-2008-1067425. 18600614 10.1055/s-2008-1067425

[52] Nolte T, Guiney J, Fonagy P, Mayers LC, Luyten P. Interpersonal stress regulation and the development of anxiety disorders: an attachment-based developmental framework. Front Behav Neurosci. 2011;5, Article 55. 10.3389/fnbeh.2011.00055. 10.3389/fnbeh.2011.00055 PMC3177081 21960962

[53] Miniati M, Callari A, Pini S. Adult attachment style and suicidality. Psychiatria Danubina. 2017;29(3):250–59. 10.24869/psyd.2017.250. 28949306 10.24869/psyd.2017.250

[54] Lai F, Wang L, Zhang J, Shan S, Chen J, Tian L. Relationship between social media use and social anxiety in college students: Mediation effect of communication capacity. Int J Environ Res Public Health. 2023;20(4):3657. 10.3390/ijerph20043657. 10.3390/ijerph20043657 PMC9966679 36834357

[55] Kim E, Cho I, Kim E. Structural equation model of smartphone addiction based on adult attachment theory: mediating effects of loneliness and depression. Asian Nurs Res. 2017;11(2):92–97. 10.1016/j.anr.2017.05.002. 10.1016/j.anr.2017.05.002 28688505

[56] Kim E, Koh E. Avoidant attachment and smartphone addiction in college students: the mediating effects of anxiety and self-esteem. Comput Hum Behav. 2018;84:264–71. 10.1016/j.chb.2018.02.037.

[57] López-Mora C, Carlo G, Roos J, Maiya S, González-Hernández J. Perceived attachment and problematic smartphone use in young people: mediating effects of self-regulation and prosociality. Psicothema. 2021;33(4):564–70. 10.7334/psicothema2021.60. 34668470 10.7334/psicothema2021.60

[58] Parent N, Bond T, Shapka J. Smartphones as attachment targets: an attachment theory framework for understanding problematic smartphone use. Curr Phychol. 2021;42:7567–78. 10.1007/s12144-021-02092-w.

[59] Liu C, Ma JL. Adult attachment style, emotion regulation, and social networking sites addiction. Front Psychol. 2019;10:2352. 10.3389/fpsyg.2019.02352 10.3389/fpsyg.2019.02352 PMC6843004 31749729

[60] Chen X, Li R, Zhang P, Liu X. The moderating role of state attachment anxiety and avoidance between social anxiety and social networking sites addiction. Psychological Rep. 2019;123(3):633–47. 10.1177/0033294118823178. 10.1177/0033294118823178 30612521

[61] Stănculescu E, Griffiths MD. Social media addiction profiles and their antecedents using latent profile analysis: The contribution of social anxiety, gender, and age. Telemat Inform. 2022;74:101879. 10.1016/j.tele.2022.101879

[62] Fonagy P, Allison E. The role of mentalizing and epistemic trust in the therapeutic relationship. Psychotherapy. 2014;51(3):372–80. 10.1037/a0036505. 24773092 10.1037/a0036505

[63] Fonagy P, Steele M, Steele H, Moran G, Higgitt A. The capacity for understanding mental states: the reflective self in parent and child and its significance for security of attachment. Infant Ment Health J. 1991;12(3):201–18. 10.1002/1097-0355(199123)12:3.

[64] Schwarzer N, Nolte T, Gingelmaier S. Mentalisierungsfähigkeit, Mentalisierungsinteresse und Persönlichkeitsdimensionen. Prävention und Gesundheitsförderung. 2022;18(2):221–27. 10.1007/s11553-022-00948-y.

[65] Fonagy P, Gergely G, Jurist EL, Target M. Affect regulation, mentalization, and the development of the self. Other Press; 2002.

[66] Mikulincer M, Shaver PR. Attachment in adulthood: structure, dynamics, and change. 2nd. Guilford; 2007.

[67] Van Loh J. The influence of media on the ability to mentalize. In: Brisch K-H, editor. Disturbed attachments in digital times. Klett-Cotta; 2023.

[68] Fehlbaum L, Borbás R, Paul K, Eickhoff S, Raschle N. Early and late neural correlates of mentalizing: ALE meta-analyses in adults, children and adolescents. Soc Cognit Affective Neurosci. 2021;17:351–66. 10.1093/scan/nsab105. 10.1093/scan/nsab105 PMC8972312 34545389

[69] Frith C, Frith U. The neural basis of mentalizing. Neuron. 2006;50:531–34. 10.1016/j.neuron.2006.05.001. 16701204 10.1016/j.neuron.2006.05.001

[70] Ding K, Shen Y, Liu Q, Li H. The effects of digital addiction on brain function and structure of children and adolescents: a scoping review. Healthcare. 2024;12(1), Article 15. 10.3390/healthcare12010015. 10.3390/healthcare12010015 PMC10779052 38200921

[71] Dong GH, Wang M, Wang Z, Zheng H, Du X, Potenza MN. Addiction severity modulates the precuneus involvement in internet gaming disorder: functionality, morphology and effective connectivity. Prog Neuro-Psychopharmacol Biol Psychiatry. 2020;98, Article 109829. 10.1016/j.pnpbp.2019.109829. 10.1016/j.pnpbp.2019.109829 31790725

[72] Goldstein RZ, Volkow ND. Dysfunction of the prefrontal cortex in addiction: neuroimaging findings and clinical implications. Nat Rev Neurosci. 2011;12(11):652–69. 10.1038/nrn3119. 22011681 10.1038/nrn3119 PMC3462342

[73] Schmitgen MM, Horvath J, Mundinger C, Wolf ND, Sambataro F, Hirjak D, et al. Neural correlates of cue reactivity in individuals with smartphone addiction. Addictive Behaviors. 2020;108, Article 106422. 10.1016/j.addbeh.2020.106422. 10.1016/j.addbeh.2020.106422 32403056

[74] Imperatori C, Corazza O, Panno A, Rinaldi R, Pasquini M, Farina B, et al. Mentalization impairment is associated with problematic Alcohol use in a sample of young adults: a cross-sectional study. Int J Environ Res Public Health. 2020;17(22):8664. 10.3390/ijerph17228664. 33266367 10.3390/ijerph17228664 PMC7700465

[75] Dozier M, Stovall-McClough KC, Albus KE. Attachment and psychopathology in adulthood. In: Cassidy J, Shaver PR, editors. Handbook of attachment: theory, research, and clinical applications. 2nd. Guilford Press; 2008. p. 718–44.

[76] Mikulincer M, Shaver PR, Mikulincer M, Shaver PR. In, Attachment in adulthood: structure, dynamics, and change. 2nd. Guilford Press; 2017. Attachment bases of psychopathology. p. 395–443.

[77] Schindler A. Attachment and substance use disorders-theoretical models, empirical evidence, and implications for treatment. Front Psychiatry. 2019;10:727. 10.3389/fpsyt.2019.00727. 31681039 10.3389/fpsyt.2019.00727 PMC6803532

[78] Kuzu Kumcu M, Törenli Kaya Z, Hoşgören Alıcı Y. Mentalizing self mind but not others: self-reported mentalization difficulties in multiple sclerosis. Brain Behav. 2024;14(7):e3612. 10.1002/brb3.3612. 10.1002/brb3.3612 PMC11226550 38970254

[79] Bersani FS, Accinni T, Carbone GA, Corazza O, Panno A, Prevete E, et al. Problematic use of the Internet mediates the association between reduced mentalization and suicidal ideation: a cross-sectional study in young adults. Healthcare (Basel, Switzerland). 2022;10(5):948. 10.3390/healthcare10050948. 10.3390/healthcare10050948 PMC9140488 35628085

[80] Lieberman A, Schroeder J. Two social lives: how differences between online and offline interaction influence social outcomes. Curr Opin Phychol. 2020;31:16–21. 10.1016/j.copsyc.2019.06.022. 10.1016/j.copsyc.2019.06.022 31386968

[81] Scott RA, Stuart J, Barber BL. Connecting with close friends online: a qualitative analysis of young adults’ perceptions of online and offline social interactions with friends. Comput Hum Behav Rep. 2022;7:100217. 10.1016/j.chbr.2022.100217.

[82] Wright PJ, Raynor PA, Bowers D, Combs EM, Corbett CF, Hardy H, et al. Leveraging digital technology for social connectedness among adults with chronic conditions: a systematic review. Digit Health. 2023;9:20552076231204746. 10.1177/20552076231204746. 37799504 10.1177/20552076231204746 PMC10548813

[83] Büchi M, Festic N, Latzer M. Digital overuse and subjective well-being in a digitized society. Soc Media + Soc. 2019;5(4). 10.1177/2056305119886031.

[84] Zhou M, Li F, Wang Y, Chen S, Wang K. Compensatory social networking site use, family support, and depression among college freshman: three-wave panel study. J Med Internet Res. 2020;22(9):e18458. 10.2196/18458. 32795999 10.2196/18458 PMC7495252

[85] Chen B, Liu F, Ding S, Ying X, Wang L, Wen Y. Gender differences in factors associated with smartphone addiction: a cross-sectional study among medical college students. BMC Psychiatry. 2017;17:341. 10.1186/s12888-017-1503-z. 29017482 10.1186/s12888-017-1503-z PMC5634822

[86] Varchetta M, Tagliaferri G, Mari E, Quaglieri A, Cricenti C, Giannini AM, et al. Exploring gender differences in Internet addiction and psychological factors: a study in a Spanish sample. Brain Sci. 2024;14(10):1037. 10.3390/brainsci14101037. 39452049 10.3390/brainsci14101037 PMC11505988

[87] Chowdhury N, Rabel EJ, Ferraz D, Del Rio Carral M. Between being affected and being an active emotion ‘manager’: young women’s accounts of social media use and wellbeing. Phychol Health. 2025;1–21. 10.1080/08870446.2025.2474011. 10.1080/08870446.2025.2474011 40045473

[88] Riedl D, Kampling H, Nolte T, Lampe A, Beutel ME, Brähler E, et al. Measuring impairments of mentalization with the 15-item mentalization questionnaire (MZQ) and introducing the MZQ-6 short scale: reliability, validity and norm values based on a representative sample of the German population. Diagnostics (Basel, Switzerland), 13(1), Article 135. 2022. 10.3390/diagnostics13010135. 10.3390/diagnostics13010135 PMC9818984 36611427

[89] Hausberg MC, Schulz H, Piegler T, Happach CG, Klöpper M, Brütt AL, et al. Is a self-rated instrument appropriate to assess mentalization in patients with mental disorders? Development and first validation of the mentalization questionnaire (MZQ). Psychother Res: J Soc Psychother Res. 2012;22(6):699–709. 10.1080/10503307.2012.709325. 10.1080/10503307.2012.709325 22867004

[90] Bian M, Leung L. Linking loneliness, shyness, smartphone addiction symptoms, and patterns of smartphone use to social capital. Soc Sci Comput Rev. 2015;33(1):61–79. 10.1177/0894439314528779.

[91] Andreassen CS, Billieux J, Griffiths MD, Kuss DJ, Demetrovics Z, Mazzoni E, et al. Bergen social media addiction Scale (BSMAS).[Database record]. APA PsycTests. 2016. 10.1037/t74607-000.

[92] Monacis L, de Palo V, Griffiths MD, Sinatra M. Social networking addiction, attachment style, and validation of the Italian version of the Bergen social media addiction Scale. J Behav Addict. 2017;6(2):178–86. 10.1556/2006.6.2017.023. 10.1556/2006.6.2017.023 PMC5520120 28494648

[93] Chen IH, Strong C, Lin YC, Tsai MC, Leung H, Lin CY, et al. Time invariance of three ultra-brief internet-related instruments: Smartphone application-based addiction Scale (SABAS), Bergen social media addiction Scale (BSMAS), and the nine-item Internet gaming disorder Scale- Short form (IGDS-SF9) (study part B). Addictive Behaviors. 2020;101:105960. 10.1016/j.addbeh.2019.04.018. 31072648 10.1016/j.addbeh.2019.04.018

[94] Santoro G, Costanzo A, Franceschini C, Lenzo V, Musetti A, Schimmenti A. Insecure minds through the looking glass: the mediating role of mentalization in the relationship between adult attachment styles and problematic social media use. Public Health. 2024;21(3):255. 10.3390/ijerph21030255. 10.3390/ijerph21030255 PMC10969895 38541257

[95] Blackwell D, Leaman C, Tramposch R, Osborne C, Liss M. Extraversion, neuroticism, attachment style and fear of missing out as predictors of social media use and addiction. Pers Individ Dif. 2017;116:69–72. 10.1016/j.paid.2017.04.039.

[96] Eroglu Y. Interrelationship between attachment styles and Facebook addiction. J Educ Train Stud. 2015;4(1):150–60. 10.11114/jets.v4i1.1081.

[97] Oldmeadow JA, Quinn S, Kowert R. Attachment style, social skills, and Facebook use amongst adults. Comput In Hum Behav. 2013;29(3):1142–49. 10.1016/j.chb.2012.10.006.

[98] Schwarzer NH, Dietrich L, Gingelmaier S, Nolte T, Bolz T, Fonagy P. Mentalizing partially mediates the association between attachment insecurity and global stress in preservice teachers. Front Phychol. 2023;14:1204666. 10.3389/fpsyg.2023.1204666. 10.3389/fpsyg.2023.1204666 PMC10475550 37671112

[99] Cheng C, Lau Y, Chan L, Luk J. Prevalence of social media addiction across 32 nations: meta-analysis with subgroup analysis of classification schemes and cultural values. Addict Behav. 2021;117, Article 106845. 10.1016/j.addbeh.2021.106845. 10.1016/j.addbeh.2021.106845 33550200

